# Protocol for the REBOUND study: a cohort study to uncover fundamental mechanisms of accelerated ageing and impaired resilience following cancer surgery and treatment

**DOI:** 10.1186/s12877-025-06109-y

**Published:** 2025-07-08

**Authors:** Carly Welch, Animesh Acharjee, Rebecca Birch, João Pedro de Magalhães, Niharika A. Duggal, Alison Hainsworth, Jose R. Hombrebueno, Simon W. Jones, Jonathan Lewis, Ali Mazaheri, Helen M. McGettrick, Thomas Nicholson, Judith Partridge, Thomas Pinkney, Claire J Steves, Kristina Tomkova, Daisy Wilson, Thomas A Jackson

**Affiliations:** 1 Department of Twin Research & Genetic Epidemiology, School of Life Course and Population Sciences, Faculty of Life Sciences and Medicine, King’s College London, 3rd Floor South Wing D Block, St Thomas’ Campus, Westminster Bridge, London, SE17EH UK; 2 Department of Ageing and Health, St Thomas’ Hospital, Guy’s and St Thomas’ NHS Foundation Trust, 9th Floor, Westminster Bridge, London, SE17EH UK; 3 Department of Cancer and Genomic Sciences, School of Medical Sciences, College of Medicine and Health, University of Birmingham, Edgbaston, Birmingham, B15 2TT UK; 4 Department of Inflammation and Ageing, School of Infection, Inflammation and Immunology, College of Medicine and Health, University of Birmingham, Edgbaston, Birmingham, B15 2TT UK; 5 Department of Colorectal Surgery, St Thomas’ Hospital, Guy’s and St Thomas’ NHS Foundation Trust, 12th Floor, Westminster Bridge, London, SE17EH UK; 6 School of Psychology, College of Life and Environmental Sciences, University of Birmingham, Edgbaston, Birmingham, B15 2TT UK; 7 Department of Applied Health Sciences, School of Health Sciences, College of Medicine and Health, University of Birmingham, Edgbaston, Birmingham, B15 2TT UK; 8 Department of Academic Surgery, Queen Elizabeth Hospital Birmingham, University Hospitals Birmingham NHS Foundation Trust, Mindelsohn Way, B152GW Edgbaston, Birmingham, UK; 9 Department of Healthcare for Older People, Queen Elizabeth Hospital Birmingham, University Hospitals Birmingham NHS Foundation Trust, Mindelsohn Way, B152GW Edgbaston, Birmingham, UK

**Keywords:** Frailty, Sarcopenia, Delirium, Stratified medicine, Senescence

## Abstract

**Background:**

Ageing is a heterogeneous process, which is associated with heterogeneous resilience in older people. Cancer surgery and treatment may be associated with accelerated ageing in some older people; studying this process will improve understanding to enable treatments to prevent adverse effects on physical and cognitive function.

**Methods:**

This study will recruit 172 participants aged 65 years and older scheduled to undergo elective colorectal surgery for cancer from two hospital sites (Guy’s and St Thomas’ NHS Foundation Trust and University Hospitals Birmingham NHS Foundation Trust). Assessments will be performed preoperatively, days 1–3 postoperatively, 30 days postoperatively, and 90 days postoperatively. These will include in-depth clinical phenotyping including handgrip strength, Short Physical Performance Battery, muscle ultrasound, cognitive tests, Electroencephalography, questionnaires including quality of life, and physical activity using remote devices. Serial blood and stool specimens will be collected across timepoints to measure underlying hallmarks of ageing including inflammation, dysbiosis, macroautophagy, cellular senescence, epigenetic alterations, mitochondrial dysfunction, and stem cell exhaustion. A machine learning approach will be utilised to evaluate the associations between trajectories in clinical and physiological parameters and fundamental biological processes.

**Discussion:**

This study represents an exciting collaboration between clinicians, fundamental scientists, and experts in machine learning. It offers the opportunity to characterise and understand complex pathways to enable future clinical trials directed towards the prevention of accelerated ageing through a stratified medicine approach.

**Supplementary Information:**

The online version contains supplementary material available at 10.1186/s12877-025-06109-y.

## Background

As we age, our biological systems and clinical features become increasingly diverse. This diversity can lead to either healthy ageing or development of age-related conditions such as frailty, sarcopenia, and cognitive decline [1]. Advancement of age is associated with reduced resilience, hindering the ability to maintain balance and stability when confronted with infections, falls, or surgery [2]. This loss of dynamic resilience with age, whilst universally recognised, is poorly defined or understood biologically, limiting our ability to intervene pharmacologically to maintain health in old age. Cancer treatment is associated with increased incidence of frailty, sarcopenia, and cognitive decline, and may lead to accelerated ageing through perturbations of pathways that are protective against cancer but predispose towards ageing.

Advances in ageing biology have enabled the description of key biological mechanisms; the hallmarks of ageing [3]: genomic instability, telomere attrition, epigenetic alterations, loss of proteostasis, disabled macroautophagy, deregulated nutrient-sensing, mitochondrial dysfunction, cellular senescence, stem cell exhaustion, altered intercellular communication, chronic inflammation, and dysbiosis. Importantly, targeting these ageing processes, for example by deleting senescent cells [4], extends healthspan and reduces development of age-related conditions in mice. Over 1000 longevity drugs and compounds have been identified in model organisms [5]. Drugs that target ageing processes to combat age-related diseases, geroprotectors, are being trialled in small scale studies in humans [6]. There is an urgent need to expand this activity to determine whether geroprotectors can also increase dynamic resilience in older humans.

However, to achieve this we need to address fundamental knowledge gaps that currently hinder such trials occurring. These gaps are:

1. Knowledge of how hallmarks of ageing influence resilience and frailty, and their response to geroprotectors. This is limited by a lack of human models to describe the clinical expression resultant of perturbed underlying biology following surgery, chemotherapy, or other stressors.
2. Lack of data demonstrating biological efficacy (effect size and duration of treatment) of key geroscience interventions in clinical populations of older people, (especially those with and without existing functional biological impairment), preventing robust trial design.
3. Lack of agreed trial outcome measures encompassing the multiple domains required to capture dynamic resilience and frailty.

Although advancing age is a risk factor for adverse outcomes after elective surgery, it has become clear that pre-existing frailty is a better prognostic indicator [6]. Thus, patients of the same chronological age do not share the same surgical risk and instead show differing levels of dynamic resilience. We hypothesise that variability in resilience is based upon varying rates of biological ageing, offering opportunities for intervention. This article presents the protocol for the “REsilience Breakthoughs in Older people UNdergoing cancer proceDures (REBOUND)” study.

## Methods

### Aim

- This study aims to identify the association of biological hallmarks of ageing with negative changes in physical and cognitive function following colorectal surgery, with or without systemic anti-cancer treatment, in older people.
- This will also enable assessment of feasibility of stratified drug trials to target hallmarks of ageing following biological stressor events.

### Design and setting

Participants will be recruited from two tertiary university hospital trusts within the UK– University Hospitals Birmingham NHS Foundation Trust, and Guy’s and St Thomas’ NHS Foundation Trust. Both of these NHS trusts serve diverse populations. Participants will be recruited prior to surgery with assessments performed within preoperative assessment clinic or Clinical Research Facilities. Follow-up assessments will be performed on the ward or intensive care unit during admission (day 1 and day 3 post-operative), and at 30- and 90-days post-surgery. Follow-up assessments may be performed at clinic appointments, Clinical Research Facilities, home visits, or through virtual review where other options are not possible.

### Characteristics of participants

Inclusion criteria are: aged 65 years and older at time of recruitment, scheduled to undergo operative management for colorectal cancer, and able to provide consent to participate at time of recruitment. Exclusion criteria are: systemic cancer treatment prior to elective surgery, or receiving end of life treatment. Prospective consent will be obtained from participants to remain in the study should they lose capacity to consent to ongoing research involvement post-operatively.

### Processes and research procedures

Table 1 shows the timing of individual research procedures that will be performed as part of this study. These include assessments of physical function, cognitive function, body composition, physical activity, and biological studies. The assessments selected within this study follow the agreed standards of the UK Geriatric Medicine Core Dataset Extended Working Group [7].

**Table 1:** – Timing of individual procedures involved in this study

	Pre-operative (Day 0)	Intra-operative	Post-operative Day 1	Post-operative Day 3	Post-operative Day 30 (+/-7)	Post-operative Day 90 (+/-10)
**All study participants** (*N*** = 172)**						
Barthel Index	X				X	X
Nottingham Extended Activities of Daily Living	X				X	X
Frailty assessment	X				X	X
Handgrip strength	X			X	X	X
Short Physical Performance Battery	X			X	X	X
Ultrasound quadriceps	X			X	X	X
Bioelectrical Impedance Analysis	X			X	X	X
Electro-encephalography (EEG)	X			X		X
Montreal Cognitive Assessment (MoCA)	X				X	X
Ecological momentary assessments	X				X (At home during study period)	X
Delirium testing			X	X		
Blood spots	X		X	X	X	X
Stool collection	X			X		X
Venepuncture	X		X		X	X
EORTC QLQ-C30	X				X	X
Three-day food record	X			X	X	X
Step count					X	X
**Selected participants**						
Muscle biopsies		X				X
Fat biopsies		X				X
Additional venepuncture	X	X				X

### Cognitive assessment

#### Montreal cognitive assessment

Cognition will be tracked serially across the study through assessment with the Montreal Cognitive Assessment (MoCA) [8] at recruitment, and Day 30- and Day 90- postoperatively. Scores within each domain of the MoCA will be recorded.

#### Delirium screening

Delirium assessment should be performed as part of routine care for all participants. However, it is recognised that this might not always be performed in practice. Therefore, the investigator should ensure that all recruited participants are screened and assessed for delirium at postoperative days 1 and 3. Participants will be screened for evidence of delirium at each visit using the 4 “As” Test (4AT). Where a possibility of delirium is raised upon screening or during other assessments, a formal assessment to assess for evidence of delirium as per DSM-V will be performed by a suitably trained clinician [9] (see Online supplement). Where delirium is present at the time of assessment, severity will be scored using the Memorial Delirium Assessment Scale [10]. Training at each site will be provided by the lead geriatrician.

#### Ecological momentary assessments

Web-based verbal fluency tests will be delivered to monitor patients’ cognitive functioning throughout the study. REDCap (secure encrypted software) will provide gentle reminders via email for the participant to complete simple memory tests/surveys. This will allow remote monitoring of the participants’ cognitive function throughout the study period.

#### Electroencephalography

Electroencephalography (EEG) recordings will be conducted using a saline-soaked 20-channel EEG net, specifically designed for a quick and gel-free application. The EEG measurements will be taken during the performance of cognitive paradigms, as well as during two periods of resting state conducted before and after the cognitive tasks. These recordings will provide valuable insights into brain activity patterns. The cognitive tasks performed during the EEG recordings will include the Attention Network Task [11] (this task will take 15 min), which has proven to be effective in studying attentional processes, and a versatile language comprehension paradigm (this task will take 1 min) [12]. These paradigms have previously shown their ability to detect subtle changes in brain function associated with cognitive impairment [13] and heightened inflammation.

### Muscle quantity and quality assessment

#### Quadriceps ultrasound

Ultrasound measurement of quadriceps muscle will be performed using two-dimensional B-mode ultrasonography with a linear probe, as previously described [14]. Participants will be positioned in a semi-upright position with their knees resting at 10–20° using a firm wedge [15]. Thickness measurements will be taken in the transverse plane at the midpoint from the greater trochanter to the lateral joint line of the knee. The Bilateral Anterior Thigh Thickness (BATT) will be calculated as the total thickness of right VI + right RF + left VI + left RF. Subcutaneous tissue thickness will also be measured. Rectus Femoris echogenicity (associated with intra-muscular fat infiltration) will be determined using grey-scale analysis on Image J software.

#### Bioelectrical impedance analysis

Bioelectrical impedance analysis (BIA) will be measured at each visit using the Bodystat Quadscan 4000. Skeletal Muscle Mass will be estimated from resistance and impedance measurements using the Sergi equation.

#### Body composition using CT imaging performed during routine medical care

Skeletal muscle and fat mass will also be measured using Computed Tomography (CT) imaging performed as part of routine clinical care. This has previously been performed by manual delineation and semi-automated measurement of cross-sectional area at the level of the third lumbar vertebra (L3) on CT in multiple cohorts. This study will build on this methodology with utilisation of a fully automated model developed from deep learning processes [16].

### Muscle and physical function assessment

#### Handgrip strength

Handgrip strength will be measured in both arms with the participant sat out in a chair, their elbow bent at 90^o^ and the forearm supinated. Participants will be advised to squeeze as hard as they can twice on each side, and the best reading will be used for analysis [17]. During hospital admission, if it is not possible for the participant to sit out in a chair, handgrip strength will be measured in bed in the most upright position possible.

#### Physical performance

The Short Physical Performance Battery (SPPB) is a standardised measure of physical performance that has been shown to be sensitive to change and provides an objective measure of physical function [18]. SPPB consists of usual gait speed, side-by-side stand, semi-tandem stand, tandem stand, and five chair stands. A total score of 12 is derived, with a lower score representing reduced physical performance.

#### Physical activity assessment

Participants will be provided with Fitbit Inspire 2 devices at the time of recruitment. They will be advised to wear this on their non-dominant wrist throughout the study period. Each device will be linked to an anonymised account to enable remote extraction of daily step count data across the study period.

### Questionnaires

#### Three day food record

Participants will be supplied with a three-day food record and advised to complete this for the three days before their appointments. This will be used to estimate daily calorific and protein intake at each assessment, adjusted for the participant’s weight at that timepoint.

#### EORTC QLQ-C30

The European Organisation for Research and Treatment for Cancer Quality of Life Questionnaire Core 30 (EORTC QLQ-C30) is one of the most commonly administered quality of life questionnaires to participants in cancer trials [19]. This will be administered at the start and end of the study.

#### Nottingham extended activities of daily living

The Nottingham Extended Activities of Daily Living (NEADL) [20] will be used to collect information on instrumental activities of daily living.

### Frailty and sarcopenia diagnoses

#### Frailty index

A frailty index will be calculated from routinely collected information and information collected in the study elsewhere, with direct questioning where necessary (Online supplement) [21].

#### Frailty phenotype

Frailty phenotype [22] will be assessed utilising information collected from assessment of handgrip strength and gait speed, and directly asking the participant about weight loss, physical activity, and self-reported exhaustion (Online supplement).

#### Clinical frailty scale

Clinical Frailty Scale (CFS) [23] will be assessed by the investigator by reviewing the clinical records and an overall holistic assessment of the participant, considering the results of the NEADL and other assessments.

#### Sarcopenia

Sarcopenia will be defined in accordance with the European Working Group on Sarcopenia in Older People 2 (EWGSOP2) definition [24]. Cut-off points are defined in accordance with previous studies and validation from young healthy reference populations (Online supplement) [21].

### Other routinely collected information

It is recognised that it should be possible to collect detailed clinical information from the medical notes without having to ask the participants specifically. Use of medical records for this purpose will be specifically explained within the consent process. Where necessary, details will be confirmed directly with the patient to ensure these are recorded correctly. This information will include cancer type and tumour stage, operative details, treatments delivered (including systemic anti-cancer treatment), and any post-operative complications. A full list of variables collected through this process has been included within the online supplement.

### Sample collection

#### Venepuncture and blood sample Preparation

Blood will be collected peripherally (or centrally if central access is in place as part of clinical care) using BD vacutainers. A maximum of 70mL of blood will be taken at any one time. Where possible, blood samples will be collected alongside blood taken as part of normal clinical care. Samples will be prepared to enable further analysis and frozen at -80^o^C (this will include whole blood, peripheral blood mononuclear cells, serum, and plasma samples).

#### Capillary blood spot collection

Capillary blood spots will be collected by utilising a lancet to prick a finger and blood collection devices will be used to collect a metered volume of whole blood. Blood will be separated into its red cell and plasma components within the device.

#### Fat, muscle, and skin biopsies

Fat, muscle, and skin samples will be collected intraoperatively from the abdominal wall by the operating surgeon. Further fat and skin samples will be collected at 90 days from the abdominal wall at follow-up through an additional procedure to their clinical care.

#### Stool sample collection

Stool samples will be collected in sterile containers and frozen at -80^o^C prior to analysis.

## Planned experimental methodology

### Stool Microbiome and metagenomics

Dysbiosis is a hallmark of ageing that influences health [2]. Microbial composition and functional potential will be assessed through metagenomics in stool to identify biomarkers of resilience using state of the art metagenomic sequencing (Illumina NovaSeq6000 DX sequencer). This will include bacterial taxonomic and functional profiling, and virus profiling [25]. This will allow assessment of the resilience of the gut microbiome to two distinct stressors and its relation to functional and cognitive resilience. This offers an opportunity for future nutritional interventions with potential for rapid clinical impact. The day three stool will be affected by the sterile inflammation of the operation, potential bowel prep, and the use of antibiotics in the immediate post-operative period. This may make longitudinal assessment between days 0, 3 and 90 difficult, but allows the comparison between degrees of change in response to the antibiotic stressors as a marker of resilience itself.

### Blood spot analysis for markers of inflammation

Blood spots will be used to track inflammation by proteomic analysis for a range of pro- and anti-inflammatory cytokines linked to frailty development [26]. Eluted samples will be analysed using the Olink Inflammatory panel and Nightingale metabolomics. Pilot data from UKBiobank show that Nightingale analytes are tightly correlated with data derived from serum, and published data show that blood spot-derived Olink data appropriately tracks clinical state in inflammatory conditions [27].

### DNA methylation, immune system ageing, compromised autophagy, mitochondrial function, nutrient sensing and stem cell exhaustion

Epigenetic age and epigenetic response (at days 0, 30 and 90) will be assessed from isolated whole blood DNA using the Illumina EPIC array and published algorithms [6]. As a novel method, the same algorithms will be run on muscle and adipose tissue samples. Immunesenescence will be assessed by deriving the IMM-AGE score [13] by immunostaining and flow cytometry analysis of isolated peripheral blood mononuclear cells identifying eight immune cell types (total T cells, naive CD4 T cells, effector memory CD4 and CD8 T cells, EMRA CD8 T cells, CD28 − CD8 T cells, CD57 + CD8 T cells and regulatory T cells). Epigenetic age and immunesenescence are associated with risk of morbidity and mortality. Immunesenescence has been shown in mice to be a key driver of frailty [28] and in humans to predict impaired resilience in patients following traumatic injury [29]. Autophagic flux assessed by LC3-II breakdown by flow cytometry will determine autophagy competence from whole blood [30]. T cells will be assessed for mTOR activation (by phospho flow cytometry) and mitochondrial function (measuring cellular bioenergetics using the Seahorse technology) as further hallmarks of ageing. Stem cell exhaustion, a hallmark of ageing, will be assessed in mesenchymal stem cells (MSCs) isolated from peripheral blood, to evaluate multipotency, senescence and metabolic plasticity.

### Adipose tissue and muscle biopsies to assess cellular senescence and tissue-based resilience biomarkers

Transcriptomics analysis (bulk RNAseq), will allow assessment of senescent cell burden using the SenMayo analysis [31], and cross-validation with the CellAge database [32]. This will be used to identify associations between dynamic resilience and canonical signalling pathways that mediate muscle mass and myogenesis (Ingenuity Pathway Analysis) and adipogenesis linked with dynamic resilience or progression of frailty. Protein coding and non-coding RNAs associated with resilience will also be assessed. Primary adipose-derived stem cells (ADSC) or primary myoblasts will be isolated from a third of the tissue and assessed immediately for autophagic flux and mitochondrial function or frozen for later use. Myoblasts will be differentiated into multinucleated myotubes using culture media to enable assessment of markers of muscle growth, differentiation, and function [33–35]. The remaining snap frozen tissue will be analysed histologically to confirm the presence of senescent cells (Lamin B1, SA-β-gal, TAF, p16, p21, γH2AX) and lipid accumulation (e.g., Oil Red O staining).

### Statistical analysis

#### Power and sample size calculation

Using data from a previous study recruiting older patients aged 70 years and older undergoing elective colorectal surgery [36], expected baseline data are: mean gait speed 0.815 m/s (SD 0.238), mean Short Physical Performance Battery (SPPB) score 8.92 (SD 2.31). In order to identify changes consistent with widely accepted minimally clinically important differences of each (0.1 m/s for gait speed [37] and 1point for SPPB [38]), a clinical trial would require a sample size of 89 for gait speed, or 84 for SPPB with 80% power and α = 0.05, or 119 or 112 respectively with 90% power and α = 0.05, in order to demonstrate clinically important differences at population level. The expected drop-out rate at 90 days from the previous study mentioned was 12.5% [39], but we accept that this can be unpredictable in this cohort and could be higher. A recruitment sample size of 172 participants (aiming for equal gender split) will enable follow-up data to 90 days for 150 participants with 12.5% drop-out, or 129 participants with 25% drop-out. This sample size should be sufficient to enable analysis of changes at population level, but also enable stratification through machine learning models [40] and multivariate associations, as set out within the planned data analysis section below.

### Planned data analysis

To determine associations of dynamic resilience within complex clinical and biological datasets a machine learning approach will be used. Integrative analysis-supervised machine learning methods, including Least Absolute Shrinkage and Selection Operator (LASSO) [41] and Elastic Net (EN) [42] regression, as well as nonparametric statistical approaches will be utilised to generate prediction models. Features selected from those models will be further investigated using graphical methods. This will allow identification of key pathways influencing outcome, and determine when to intervene, i.e., pre or post challenge. Further detail of the analysis plan that will be employed is detailed below:

#### Identification of key resilience features

This will test the associations between multiple clinical outcomes (E.g., physical function, cognition) with biological measurements (E.g., transcriptomics, proteomics, hallmarks etc.). A two-step approach will be utilised. In the first step, a variety of supervised machine learning methods will be employed, including LASSO and EN to generate different prediction models, either classification or regression model (based on the clinical outcome variable). Using multiple methods will also provide methodological validation. For the application of the LASSO and EN algorithms, and the subsequent selection of the relevant features associated with the outcome variable, the penalty parameter associated with each of the methods will be optimised in an unbiased manner. To achieve this, samples will be randomly divided into training (75% samples) and test (25% samples) sets. A 10-fold cross validation will then be applied on the training set to obtain an optimised penalty parameter that can be used across the LASSO and EN models. The process will be iterated 100 times and the features appearing more frequently than a set threshold number of times (the first upper quantile) will be selected to ensure features are important and can be used for further association analysis. This step will provide candidate markers that need to be tested in the experimental set up. This step will also provide a relatively small set of the biological features compared to the entire input data sets.

#### Multivariate associations

This step is complementary to the objective above. The main difference is that here all the data will be used as a matrix of all multiple clinical outcomes for example physical function, cognition, and muscle function as a whole considering correlation structure of the clinical features. Canonical correlation analysis (CCA) [43] and related methods will be suitable for such analysis to describe the relationship between different outcome trajectories. The main goal of CCA is to identify the underlying relationships or patterns between two sets of variables (for example: Clinical vs. transcriptomics), also known as canonical variates. The hyperparameters associated with this method will be optimised using cross validation and compared with the previous step for the stability of the results across multiple methods.

#### Network analysis and visualisation

In this step, the best features based on the coefficients or weights associated with the clinical outcome variable in the previous steps will be selected. These will be utilised to generate a spearman correlation based integrated network where each node represents either a gene, protein, or immune parameter that was selected from the previous process, and the edges represent interactions or associations amongst them. A statistical significance threshold (FDR corrected) probability value will be chosen as 0.05 to draw the interactions among the nodes. Associations will be further prioritised based on the network statistics [44] and parameters such as degree, centrality, and betweenness.

#### Biological interpretation using pathway analysis

The set of genes, proteins, immune and epigenetic markers identified will be used in further pathway analysis using KEGG and Wiki pathways. Gene ontology-based analysis will be performed in addition to ensure that markers are helping to understand the mechanistic basis of dynamic resilience.

## Discussion

This study offers an exciting opportunity to fully characterise the associations of clinical manifestations of impaired resilience with underlying fundamental biological processes. The greatest strengths of this study lie in the multifaceted approach that will be utilised. This study represents a collaboration between clinicians, fundamental scientists, and experts in machine learning. Additionally, the study represents a collaboration across two universities with investment in infrastructure for translational health research, with recruitment of participants from the affiliated NHS trusts. The populations served by the two NHS trusts are geographically distinct within the UK, but each represent diverse populations in terms of both ethnicity and levels of social deprivation.

Geroprotectors offer a novel approach to treat negative consequences of ageing. Targeting treatments towards fundamental ageing pathways offers the opportunity to treat and prevent multiple long-term conditions at once, reducing healthcare expenditure and polypharmacy. This study will provide detailed clinical and biological information to enable development of stratified medicine approaches to treatment. The identification of perturbed pathways will then enable early phase drug trials to test the efficacy of drug targets against these pathways. Whilst there is a recognised need for trials of geroprotectors within clinical populations, the detailed characterisation that this study will offer will ensure that future drug trials have the greatest chance of success. Such trials can be conducted in a way that is most likely to yield patient benefit, whilst also being cost effective and preventing unnecessary risks to patients.

The 90 day follow-up period selected within this study is considered a timeframe that will enable assessment of the effects of the stressors following a period of stabilisation and recovery. It is expected that half of all study participants will undergo post-operative chemotherapy; assessing the effects of this will enable characterisation of further loss of resilience after chemotherapy. The timeframe selected within this cohort is consistent with the timeframe selected in a previous study characterising acute changes in muscle quantity and function in hospitalised older people [36], as well as other international studies. Importantly, a 90 day follow-up period could be directly translated within the design of an interventional clinical trial.

This study is investigator-led and the idea and protocol for this study have been developed solely by the authors, with the University of Birmingham acting as the study sponsor. However, another strength of this study relates to its central funding by the Wellcome Leap Dynamic Resilience programme, which is a venture jointly funded by Wellcome leap (founded by the Wellcome trust) and the Temasek trust. This is an innovative funding programme that has enabled collaboration with other researchers within the field from the outset, with other research projects working across the spectrum from discovery science to clinical trials. This early collaboration will enable the sharing of knowledge and protocol amendments should other studies identify new significant findings whilst this study is ongoing. In addition, the global collaboration within the programme will enable rapid dissemination of findings internationally upon study completion. The clinical variables selected within this study have been chosen considering the guidance of the UK Geriatric Medicine Core Dataset Working Group, which followed a Delphi approach [7]. Ensuring that variables meet these standards will enable data sharing and collaboration with external researchers.

However, we recognise that there will be some limitations to our study. To examine the effects of Dynamic Resilience to stressor responses there are many different populations that could be assessed, and there are benefits and limitations to each. The inclusion of an elective population enables pre-insult measurements to be taken prior to treatment, and thus to assess the impact of surgery and systemic anti-cancer treatment. Colorectal cancer is common amongst older people with an equal incidence amongst genders and is less commonly associated with cancer cachexia at presentation. Nevertheless, we recognise that the cancer itself may have systemic effects upon inflammation and the immune system. Thus, we cannot be certain how the effects encountered within this population can be extrapolated into other populations.

Secondly, whilst we consider our sample size to be modest and we have planned a robust data analysis plan, we recognise that the immense number of datapoints that will be recorded as part of this study is likely to mean that the interpretation of our dataset will be especially complex. We anticipate that there will be significant overlap between pathways and there are likely to be multiple biological pathways implicated within overlapping clinical manifestations. However, the results of our study can be utilised to guide further complex power calculations to support clinical trials, which may still measure fundamental biological pathways.

## Conclusions

The REBOUND study is an innovative multi-centre study across two diverse populations in the UK. The study aims to characterise how clinical manifestations of impaired resilience (including delirium, cognitive spectrum disorders, sarcopenia, functional decline, and recovery from illness) relate to underlying biological pathways of impaired resilience. Our study will offer unique insights as a model of accelerated ageing in the context of cancer treatment that will be directly relevant to older people receiving cancer treatment, but that will also have far-reaching implications on the understanding of the ageing process in general. The results of this trial can be directly utilised to develop a protocol for a clinical trial to target impaired resilience using a stratified medicine approach.

## Electronic supplementary material

Below is the link to the electronic supplementary material.

Supplementary Material 1

## Data Availability

No datasets were generated or analysed during the current study.
