# Supplementary material for: Protocol for the REBOUND study: a cohort study to uncover fundamental mechanisms of accelerated ageing and impaired resilience following cancer surgery and treatment
Source: BMC Geriatr. 2025 Jul 8;25:502. doi: 10.1186/s12877-025-06109-y (PMC12235963; doi:10.1186/s12877-025-06109-y)
Supplement: Supplementary file 1 — Supplementary Material 1 [file 12877_2025_6109_MOESM1_ESM.docx]

# Protocol for the REBOUND study: A cohort study to uncover fundamental mechanisms of accelerated ageing and impaired resilience following cancer surgery and treatment.

Online supplement

Contents

[1. Variables to be included within Frailty Index 2](#_Toc159240027)

[2. Frailty phenotype definition 4](#_Toc159240028)

[3. Sarcopenia definition 5](#_Toc159240029)

[4. Additional variables collected from routine clinical care 6](#_Toc159240030)

[Supplement references 9](#_Toc159240031)

## Variables to be included within Frailty Index

| **Deficit** | **Definition** |
| --- | --- |
| Activity limitation | Positive Fried physical activity score |
| Anaemia and haematinic deficiency | Female Hb<115, Male Hb<135, on medication for haematinic deficiency, or new haematinic deficiency identified during admission |
| Arthritis | Patient reported (includes osteoarthritis and inflammatory arthritis) |
| Atrial fibrillation | Any history – paroxysmal, temporary, or permanent |
| Cerebrovascular disease | Vascular dementia or stroke disease |
| Chronic kidney disease | eGFR <60 |
| Diabetes mellitus | Known history/ confirmed diagnosis |
| Dizziness | Patient reported |
| Dyspnoea | Patient reported |
| Falls | Two or more over previous year |
| Foot problems | Patient reported |
| Fragility fracture | Previous history |
| Hearing impairment | Need for hearing aids |
| Heart failure | Known history/ confirmed diagnosis |
| Heart valve disease | Known history |
| Housebound | Nottingham extended ADLs |
| Hypertension | On treatment or recorded |
| Presyncope/ syncope | Patient reported |
| Ischaemic heart disease | Known history |
| Memory and cognitive problems | Any cognitive spectrum disorder including mild cognitive impairment, delirium, and dementia |
| Osteoporosis | On treatment or known history |
| Parkinsonism and tremor | Includes tremor of any cause – known history or on treatment |
| Peptic ulcer | Known history |
| Peripheral vascular disease | Known history |
| Polypharmacy | ≥5 prescribed medications |
| Requirement for care | Formal carers |
| Respiratory disease | Any history of chronic disease e.g. asthma, COPD |
| Skin ulcer | Any current skin ulcer including arterial, venous, or pressure-related |
| Sleep disturbance | Patient reported |
| Social vulnerability | Lives alone |
| Thyroid disease | Known history |
| Urinary or faecal incontinence | Barthel index |
| Urinary system disease | Known history |
| Visual impairment | Wears glasses/ visual aids or on treatment for eye condition(s) |
| Weight loss and anorexia | Fried weight loss |

## Frailty phenotype definition

| Criteria | Criteria to score positive | | Source |
| --- | --- | --- | --- |
| Handgrip strength (kg) | Male | Female | Original study (1) |
|  | BMI <= 24: <=29  BMI 24-26: <=30  BMI 26-28: <=30  BMI >28: <=32 | BMI <=24: <=17  BMI 24-26: <=17.3  BMI 26-28: <=18  BMI >28: <=21 |  |
| Gait speed (m/s) | Male | Female | Original study (1) |
|  | <=1.73m height: <=0.65  >1.73m height: <=0.76 | <= 1.59m height: <= 0.65  >1.59m height: <=0.76 |  |
| Self-reported exhaustion | Answers “most of the time” or “all of the time” to the following questions.  How often over the last week have you felt that the following statements were true:   - “I felt that everything I did was an effort” - “I could not get getting” | | Original study (1) |
| Weight loss | 4.5kg OR 5% total body weight loss over last year | | Original study (1) |
| Physical activity | How often do you engage in activities that require a low or moderate level of energy such as gardening, cleaning the car, or doing a walk?   - More than once a week - Once a week - One to three times a month - Hardly ever or never | | SHARE-FI (2) |

## Sarcopenia definition

Cut-off values used for sarcopenia diagnosis. Cut-off values for handgrip strength, SMMSergi, gait speed, and SPPB are taken from those recommended by the European Working Group in Older People 2 (3). Cut-off values for BATT are taken from those recommended by Wilson et al (4).

BATT=Bilateral Anterior Thigh Thickness; SMMSergi=Skeletal Muscle Mass (Sergi equation).

|  | **Male** | **Female** |
| --- | --- | --- |
| No sarcopenia | 1. Handgrip strength ≥27kg | 1. Handgrip strength ≥16kg |
| Probable sarcopenia | 1. Handgrip strength <27kg | 1. Handgrip strength <16kg |
|  | 2. BATT ≥5.44cm AND SMMSergi ≥20kg | 2. BATT ≥3.85cm AND SMMSergi ≥20kg |
| Definite sarcopenia, not severe | 1. Handgrip strength <27kg | 1. Handgrip strength <16kg |
|  | 2. BATT <5.44cm AND/OR SMMSergi <20kg | 2. BATT <3.85cm AND/OR SMMSergi <15kg |
|  | 3. Gait speed >0.8m/s AND SPPB >8 | 3. Gait speed >0.8m/s AND SPPB >8 |
| Definite sarcopenia, severity unclear | 1. Handgrip strength <27kg | 1. Handgrip strength <16kg |
|  | 2. BATT <5.44cm AND/OR SMMSergi <20kg | 2. BATT <3.85cm AND/OR SMMSergi <15kg |
| 3. Gait speed not measured AND SPPB not measured | 3. Gait speed not measured AND SPPB not measured | |
| Definite sarcopenia, severe | 1. Handgrip strength <27kg | 1. Handgrip strength <16kg |
|  | 2. BATT <5.44cm AND/OR SMMSergi <20kg | 2. BATT <3.85cm AND/OR SMMSergi <15kg |
|  | 3. Gait speed ≤0.8m/s OR SPPB ≤8 | 3. Gait speed ≤0.8m/s OR SPPB ≤8 |

## Additional variables collected from routine clinical care

| **Domains** | **Variables** |
| --- | --- |
| Demographics | - Age - Gender - Sex - Ethnicity - Deprivation index (derived from postcode) |
| Social | - Place of residence - Need for formal and informal care at home - Household members |
| Lifestyle | - Alcohol use - Smoking history - Use of recreational drugs |
| Cancer-related | - Cancer type and histology - Tumour stage - Presence of positive biomarkers - Details of any systemic anti-cancer treatment (regime, dosage, timing) |
| Operative | - Type of operation - Duration of operation - Intra-operative blood loss - Type of anaesthetic agent - Post-operative critical care admission |
| Treatments | - Antibiotics during admission or follow-up - Steroids during admission or follow-up - Any other new medications deemed relevant |
| Nutritional | - Use of artificial feeding methods - Use of oral nutritional supplementation - Other nutritional intervention e.g., food fortification - Height, weight, and any other anthropometry measurements at each timepoint |
| Complications | - Surgical complications e.g., surgical site infection, wound dehiscence, anastomotic leak, need for further surgery - Medical complications e.g., falls, arrhythmias, infections – pulmonary, urinary, or other - Evidence of delirium documented anywhere within medical documentation (even if not present at the time of assessment by the research team below) - Unplanned critical care admission - Death during follow-up period - Readmission during follow-up period |
| Vital signs at assessment timepoints | - Systolic and diastolic blood pressure - Heart rate - Temperature - Respiratory rate - Oxygen saturations and need for supplemental oxygen - Capillary blood glucose |
| Long-term conditions | - Dementia (known diagnosis) - Stroke (any previous symptomatic disease) - Ischaemic Heart Disease - Diabetes Mellitus - Any other treated or untreated cancer within the last five years - Congestive cardiac failure - Chronic Obstructive Pulmonary Disease - Parkinsonian syndromes - Hypertension - Depression - Other relevant medical problems |
| Medications | - Drug count - Full medication history - Use of any over the counter medications or nutritional supplements |
| Laboratory test results at assessment timepoints | - Haemoglobin - Mean cell volume - Red cell distribution width - White cell count - Neutrophil and lymphocyte counts - Creatinine - Albumin - Alkaline phosphatase - Alanine transaminase - CRP |
| Others | - Length of stay - Additional routinely collected clinical information may be collected if this is considered relevant to the main study or any sub-studies arising, provided patient care is not altered by this |

## Supplement references

1. Fried LP, Tangen CM, Walston J, Newman AB, Hirsch C, Gottdiener J, et al. Frailty in older adults: evidence for a phenotype. The journals of gerontology Series A, Biological sciences and medical sciences. 2001;56(3):M146-56.

2. Romero-Ortuno R, Walsh CD, Lawlor BA, Kenny RA. A Frailty Instrument for primary care: findings from the Survey of Health, Ageing and Retirement in Europe (SHARE). BMC Geriatrics. 2010;10(1):57.

3. Cruz-Jentoft AJ, Bahat G, Bauer J, Boirie Y, Bruyère O, Cederholm T, et al. Sarcopenia: revised European consensus on definition and diagnosis. Age and ageing. 2019;48(1):16-31.

4. Wilson DV, Moorey H, Stringer H, Sahbudin I, Filer A, Lord JM, Sapey E. Bilateral Anterior Thigh Thickness: A New Diagnostic Tool for the Identification of Low Muscle Mass? Journal of the American Medical Directors Association. 2019;20(10):1247-53.e2.
